# Implementation of *CYP2D6* copy-number imputation panel and frequency of key pharmacogenetic variants in Finnish individuals with a psychotic disorder

**DOI:** 10.1038/s41397-022-00270-y

**Published:** 2022-02-23

**Authors:** Katja Häkkinen, Johanna I. Kiiski, Markku Lähteenvuo, Tuomas Jukuri, Kimmo Suokas, Jussi Niemi-Pynttäri, Tuula Kieseppä, Teemu Männynsalo, Asko Wegelius, Willehard Haaki, Kaisla Lahdensuo, Risto Kajanne, Mari A. Kaunisto, Annamari Tuulio-Henriksson, Olli Kampman, Jarmo Hietala, Juha Veijola, Jouko Lönnqvist, Erkki Isometsä, Tiina Paunio, Jaana Suvisaari, Eija Kalso, Mikko Niemi, Jari Tiihonen, Mark Daly, Aarno Palotie, Ari V. Ahola-Olli

**Affiliations:** 1 Department of Forensic Psychiatry, Niuvanniemi Hospital, University of Eastern Finland, FI-70240 Kuopio, Finland; 2 Institute for Molecular Medicine Finland (FIMM), HiLIFE, University of Helsinki, FI-00014 Helsinki, Finland; 3 Department of Clinical Pharmacology, University of Helsinki, FI-00014 Helsinki, Finland; 4 Individualized Drug Therapy Research Program, Faculty of Medicine, University of Helsinki, FI-00014 Helsinki, Finland; 5 Department of Psychiatry, University of Oulu, FI-90014 Oulu, Finland; 6 Tampere University, FI-33014 Tampere, Finland; 7 Social Services and Health Care Sector, City of Helsinki, FI-00099 Helsinki, Finland; 8 University of Helsinki, Helsinki University Hospital, Psychiatry, FI-00029 Helsinki, Finland; 9 Mental Health Unit, Finnish Institute for Health and Welfare, Helsinki, FI-00271 Finland; 10 Department of Psychiatry, University of Turku and Turku University Hospital, FI-20521 Turku, Finland; 11 Mehiläinen, FI-00260 Helsinki, Finland; 12 Department of Psychology and Logopedics, Faculty of Medicine, University of Helsinki, FI-00014 Helsinki, Finland; 13 Department of Psychiatry, Pirkanmaa Hospital District, FI-33521 Tampere, Finland; 14 Department of Psychiatry, Oulu University Hospital, FI-90220 Oulu, Finland; 15 Department of Psychiatry, University of Helsinki, FI-00014 Helsinki, Finland; 16 Pain Clinic, Department of Anesthesiology, Intensive Care and Pain Medicine, University of Helsinki and Helsinki University Hospital, FI-00290 Helsinki, Finland; 17 Department of Clinical Pharmacology, HUS Diagnostic Center, Helsinki University Hospital, FI-00029 Helsinki, Finland; 18 Department of Clinical Neuroscience, Karolinska Institutet, SE-17177 Stockholm, Sweden; 19 Center for Psychiatry Research, Stockholm City Council, SE-11364 Stockholm, Sweden; 20 Stanley Center for Psychiatric Research, The Broad Institute of MIT (Massachusetts Institute of Technology) and Harvard, MA-02142 Cambridge, MA USA; 21 Analytical and Translational Genetics Unit, Massachusetts General Hospital, MA-02114 Boston, MA USA

**Keywords:** Medical research, Genetics research

## Abstract

We demonstrate that *CYP2D6* copy-number variation (CNV) can be imputed using existing imputation algorithms. Additionally, we report frequencies of key pharmacogenetic variants in individuals with a psychotic disorder from the genetically bottle-necked population of Finland. We combined GWAS chip and *CYP2D6* CNV data from the Breast Cancer Pain Genetics study to construct an imputation panel (*n* = 902) for *CYP2D6* CNV. The resulting data set was used as a *CYP2D6* CNV imputation panel in 9262 non-related individuals from the SUPER-Finland study. Based on imputation of 9262 individuals we confirm the higher frequency of CYP2D6 ultrarapid metabolizers and a 22-fold enrichment of the *UGT1A1* decreased function variant rs4148323 (UGT1A1*6) in Finland compared with non-Finnish Europeans. Similarly, the *NUDT15* variant rs116855232 was highly enriched in Finland. We demonstrate that imputation of *CYP2D6* CNV is possible and the methodology enables studying *CYP2D6* in large biobanks with genome-wide data.

## Introduction

Pharmacogenetics is a research field studying how interindividual genetic differences contribute to drug efficacy and safety, aiding physicians in drug selection and dose adjustment [1]. Variants in *CYP2D6* and *CYP2C19* genes have been shown to affect the metabolism of antidepressants, antipsychotics, analgesics such as codeine and tramadol and the antiplatelet agent clopidogrel, for example [2–5]. Research of the *CYP2D6* locus at 22q13.2 has been limited by its complexity [6]. In addition to single nucleotide polymorphisms (SNPs), *CYP2D6* copy-number variations (CNVs), such as duplications and deletions, also contribute to the metabolic activity of the CYP2D6 enzyme. The frequency of the *CYP2D6* gene duplication is highly dependent on the population [7, 8]. The effects of genetic variants on drug metabolism are large enough to cause the Food and Drug Administration (FDA) and the European Medicines Agency (EMA) to add pharmacogenetic information on drug labels [9, 10] and the American Psychiatric Association to inform clinicians on pharmacogenetics in recently updated schizophrenia guidelines [11]. However, pharmacogenetic research has suffered from small sample sizes, sometimes due to complexity of CYP2D6 locus.

Genotype imputation has been successfully used in genome-wide association studies (GWAS) to acquire data on ungenotyped markers, facilitate fine-mapping, and boost power in association studies [12]. The method relies on the haplotype structure of the human genome. Haplotypes are block-like regions of DNA and within these blocks certain variants tend to co-occur allowing for separation of maternally and paternally inherited variants. Information of which variants are located on the same chromosome is needed to accurately impute missing variants. This information also has an important role in pharmacogenetics since it allows to infer whether, for example, two loss-of-function variants in the same gene are located on the same chromosome or not. The process of computationally separating the variants to different chromosomes is called phasing [13]. Usually, only SNPs or short indels are imputed, but since *CYP2D6* duplications and deletions tend to co-occur with certain SNPs, we hypothesized that existing imputation algorithms could be exploited to impute *CYP2D6* CNVs to a large set of GWAS chip-genotyped individuals. Usually, CNVs are genotyped with real-time PCR as *CYP2D6* CNVs are too small (4 kilobases) to be detected from GWAS chip signal intensity data. Successful imputation would allow for genotyping only a subset of the sample (a few hundred individuals), after which the CNV carrier status could be predicted in silico for hundreds of thousands of individuals. This would result in cost-effective pharmacogenetic analyses in biobanks and other large GWAS chip- genotyped samples, such as FinnGen (www.finngen.fi).

Next generation sequencing has become increasingly popular during the recent years. Several algorithms already exist to predict *CYP2D6* carrier status from sequencing depth, which is a measure of overlapping reads in a given region of the genome. Duplicated genome regions tend to have a higher sequencing depth compared with the regions of the reference genotype as more reads are generated from the duplicated region. However, sequencing data are available from smaller sample sets compared with GWAS chip data. Furthermore, *CYP2D6* CNV algorithms relying on sequencing depth tend to provide discordant CNV calls [14].

Due to the small founder population, bottleneck effects and genetic drift, the Finnish population is enriched for certain low frequency (0.5–5%) variants, including coding and loss-of-function variants [15]. Thus, our secondary aim in this work was to assess whether the unique population history has caused alterations in the frequency of pharmacogenetic variants compared with other European populations.

## Material and methods

**Fig. 1:**
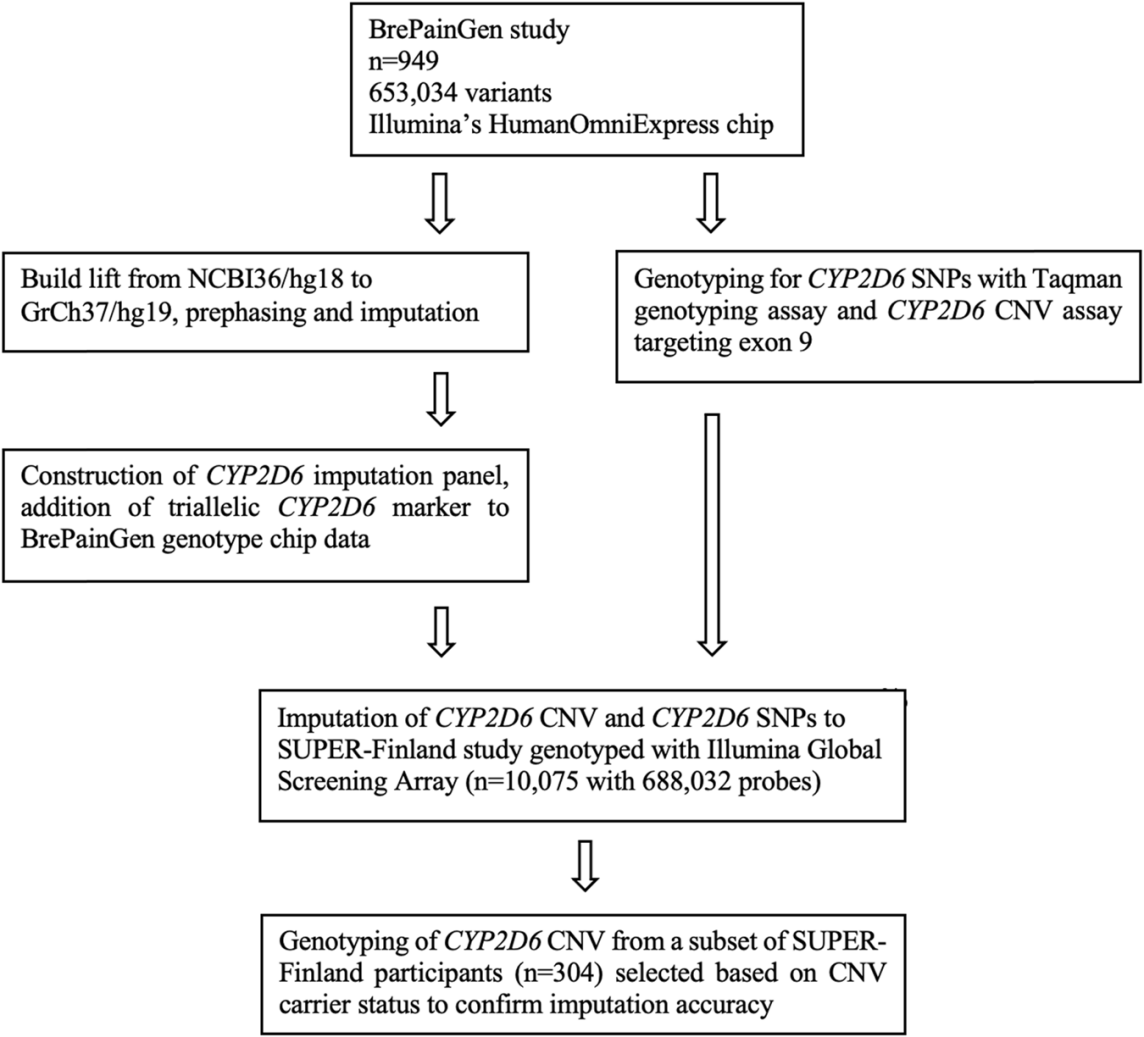
A flow chart of study protocol.

A flow chart of the study protocol is provided in Fig. 1.

### SUPER-Finland study

The SUPER-Finland study recruited 10,474 participants aged >18 with a severe mental disorder between the years 2016–2018 from Finland. Subjects were recruited from in- and outpatient psychiatric, general care, and housing units with a diagnosis of a schizophrenia spectrum psychotic disorder (ICD-10 codes F20, F22–29), bipolar disorder (F30, F31) or major depressive disorder with psychotic features (F32.3 and F33.3). As Finland contains internal genetic subisolates, special care was taken to ensure wide coverage of known isolate areas.

Blood samples were drawn from participants for DNA extraction (2x Vacutainer EDTA K2 5/4 ml, BD). When venipuncture was not possible, saliva sample (DNA OG-500, Oragene) was collected for DNA extraction. All samples were frozen (−20 °C) on site within 60 min of sampling and sent to the THL (Finnish Institute for Health and Welfare) Biobank within 3 months for long term storage in −185 °C. DNA was extracted from EDTA-blood tubes using PerkinElmer Janus chemagic 360i Pro Workstation with the CMG-1074 kit. After incubation in +50 °C, o/n DNA was extracted from saliva samples with Chemagen Chemagic MSM I robot with CMG-1035–1 kit. DNA samples were shipped on dry ice to the Broad Institute of MIT and Harvard, Boston Cambridge, Massachusetts, USA for genotyping and sequencing.

ICD-code diagnosis and disease duration (as years from receiving the diagnosis until recruitment) of the SUPER-Finland participants were extracted from The Care Register for Health Care [16].

### Genotyping and sequencing

10,075 SUPER-Finland individuals were genotyped with Illumina Global Screening Array containing 688,032 probes. Genotyping was performed at Broad Institute in Cambridge, Massachusetts, USA. Subjects with a genotyping success rate <90% and discordance between reported gender and genotyped sex were excluded. After this, variants with over 90% of missing genotype calls and related samples using pi-hat cut-off of 0.15 were excluded. Variants deviating from Hardy-Weinberg equilibrium were excluded (*P* < 1 × 10^−8^). Samples with low or excess heterozygocity (±3SD from sample mean) were excluded. Imputation was performed using the FinnGen-style imputation protocol as described in protocols.io (https://www.protocols.io/view/genotype-imputation-workflow-v3-0-nmndc5e?version_warning=no). Sequencing Initiative Suomi (SISu) v2 panel was used as the imputation reference [17].

### Construction and validation of *CYP2D6* CNV imputation panel

A *CYP2D6* CNV imputation panel was constructed using data from the Breast Cancer Pain Genetics Study (BrePainGen [18, 19]). The Finnish BrePainGen consisted of 1000 patients recruited between 2006–2010, who underwent surgery for breast cancer at the Helsinki University Hospital. The BrePainGen subjects were genotyped with HumanOmniExpress-12v1_H chip manufactured by Illumina. Before quality control, we had data consisting of 949 samples and 733,202 probes. Probes with >3% missing data were excluded (222 probes failed). After this, samples with over 3% missingness rate (0 excluded) were filtered. Variants with minor allele frequency below 0.5% were excluded (63,918 variants excluded). Subsequently, variants with Hardy-Weinberg *p* value < 1 x 10E-6 were excluded (10,857 fails). Sex check was not performed as all samples were from female study participants. In the heterozygosity check, 14 samples failed. Five samples were excluded due to relatedness. Based on MDS plots, 4 individuals were excluded. The final data set consisted of 653,034 variants and 926 samples. The genotyping rate was 0.9968. The initial dataset was lifted from NCBI36/hg18 to GrCh37/hg19 using Will Rayner’s method (https://www.well.ox.ac.uk/~wrayner/strand/). *CYP2D6* CNV was genotyped separately with real-time PCR [19]. Data on CNV were converted with R to Plink ped- and map-file format. This was then joined to the quality controlled GWAS chip data with Plink’s (version 1.9) --merge option. Next, the data were pre-phased with Eagle [20] version 2.4 and imputed with Beagle [21] version 4.1 software. The software code for CNV imputation pipeline is available upon reasonable request.

### Genotyping of *CYP2D6* CNV in a subset of SUPER-Finland participants

To confirm the copy-number imputation results, 317 SUPER-Finland subjects were selected for *CYP2D6* CNV genotyping based on CNV and SNP imputation results to guarantee a sufficient number of deletion and duplication carriers in addition to *4, *10 and *41 carriers in the validation set. Samples with discrepant CNV results in real-time PCR genotyping were excluded (*n* = 13) resulting 304 samples with imputed and genotyped CNV data. Real-time PCR genotyping was performed on QuantStudio™ 12 K Flex system (Thermo Fisher Scientific, Waltham, MA) with three TaqMan^®^ Copy Number Assays: Hs00010001_cn targeting exon 9, hs04502391_cn targeting intron 6, and hs04083572_cn targeting intron 2 of *CYP2D6* gene. Four replicates of each sample were genotyped. The reaction volume was 10 μl and RNase P was used as a reference assay. The copy-number for each sample was calculated with the CopyCaller^™^ Software (Applied Biosystems^®^) according to the manufacturer’s instructions. SNP genotyping was carried out with TaqMan OpenArray^®^ system on QuantStudio 12 K Flex real-time PCR equipment (ThermoFisher Scientific, Waltham, MA) following manufacturer´s instructions. Custom made TaqMan OpenArray^®^ included 12 clinically relevant *CYP2D6* variants defining *2, *3, *4, *6, *9, *10, *17, *29, *35, *41 and *59 alleles. Genotyping was repeated for samples with several undetermined genotypes. *CYP2D6* CNV and allele frequencies of the SUPER-Finland subset samples used for imputation panel validation are included in Supplementary Table 1.

### Haplotype construction according to CPIC guidelines

To predict pharmacogenetic phenotypes from genotype, functional annotations described in The Clinical Pharmacogenetics Implementation Consortium (CPIC) guidelines were followed for variants in the *CYP2C9* [22]*, CYP2C19* [23]*, CYP2D6* [4]*, DPYD* [24]*, NUDT15* [25]*, SLCO1B1* [26]*, TPMT* [25] and *UGT1A1* [27] genes. Variants included in the phenotype prediction from the SUPER-Finland data are described in Supplementary Table 2. If an individual did not carry any of these variants, but had been genotyped, the predicted phenotype was defaulted to normal.

### Statistical analyses

To provide estimates for the accuracy of the imputation method, we calculated sensitivity, specificity, negative predictive value (NPV) and positive predictive value (PPV), as described earlier [28]. To calculate the differences in the geographical distribution of the predicted pharmacogenetic phenotypes, we used Fisher’s test. All statistical analyses were performed in R version 3.5.1 [29].

### Ethics

The SUPER-Finland study was given a favorable ethics statement (202/13/03/00/15) by the Coordinating Ethics Committee of the The Hospital District of Helsinki and Uusimaa (HUS). The BrePainGen study was approved by the Coordinating Ethics Committee (136/E0/2006) and the Ethics Committee of the Department of Surgery (Dnro 148/E6/05) of the Hospital District of Helsinki and Uusimaa (HUS). Written informed consent was obtained and archived from each participant prior to inclusion and individual-level data was de-identified in both of the studies.

## Results

9262 non-related individuals participating in the SUPER-Finland study passed genotype quality control steps and thus had imputed genotype data available, 49.6% of these samples were female. The descriptive statistics of the sample are shown in Table 1.

**Table 1:** Characteristics of study participants (total *n* = 9262) in SUPER-Finland.

Gender % (*n*)	
Women	49.6 (4589)
Men	50.4 (4660)
Unknown	0.1 (13)
Age mean (SD)	46.6 (14.8)
Recruitment region % (*n*)	
Helsinki district (Southern Finland)	25.2 (2329)
Tampere district (Central Finland)	24.8 (2298)
Kuopio district (Eastern Finland)	20.4 (1886)
Oulu district (Northern Finland)	18.7 (1736)
Turku district (Western Finland)	10.9 (1013)
Diagnosis % (*n*)	
Schizophrenia	55.4 (5117)
Bipolar disorder (I & II)	15.4 (1419)
Other psychosis	10.1 (934)
Schizoaffective disorder	9.1 (837)
Psychotic depression	5.2 (475)
Other mental disorder	4.8 (447)
Unknown	0.4 (33)
Disease duration (years) mean (SD)	
Schizophrenia	21.9 (12.8)
Bipolar disorder (I & II)	13.7 (9.0)
Other psychosis	15.2 (11.6)
Schizoaffective disorder	21.8 (12.1)
Psychotic depression	14.2 (9.0)

### *CYP2D6* CNV imputation panel

The performance of the *CYP2D6* CNV imputation panel was evaluated by genotyping CNVs with real-time PCR from 304 SUPER-Finland subjects. Sensitivity, specificity, PPV and NPV are reported in Supplementary Table 3. The contingency table of imputed and PCR-genotyped *CYP2D6* CNVs in SUPER-Finland is presented in Table 2. Because subjects were selected based on expected copy-number for validation, NPV and PPV do not represent the situation in the general population or in the patients with psychosis as PPV and NPV are dependent on CNV frequency. The imputation method was able to identify all except two true duplication carriers as having *CYP2D6* duplication but it misclassified additional 12 subjects as having CN = 3 although these 12 subjects did not carry duplications. This results in high sensitivity (0.986) for detecting duplications. *CYP2D6* deletions were imputed correctly for 32 individuals. The method misidentified 4 individuals carrying a deletion as either having a normal copy-number or carrying a duplication (false negatives). Overall, the method showed good accuracy as *CYP2D6* CNV was imputed correctly for 278 (91.5%) individuals.

**Table 2:** A contingency table of imputed and real-time PCR genotyped *CYP2D6* copy-number (CN) in SUPER-Finland (*n* = 304).

		Imputed copy-number		
		CN = 1	CN = 2	CN = 3
Genotyped copy-number	CN = 1	32	3	1
	CN = 2	9	172	11
	CN = 3	0	1	74
	CN = 4	0	0	1

The Table 2 includes 76 subjects who has at least one duplicated *CYP2D6* gene (copy-number ≥3). Of these, 62 subjects are ultrarapid metabolizers (UMs) based on imputation. Out of the 62 subjects, 55 have both imputation and genotyping result available. For 52 subjects, we can confirm the imputation-based phenotype with genotyping. The remaining 3 individuals are either normal metabolizers (NMs) or UMs, depending on which star allele is duplicated. Thus, according to the most pessimistic view (assuming the 3 individuals were NMs), the imputation is correct for 94.5% of the samples. For the 304 subjects in the Table 2 the corresponding percentages for NMs, intermediate metabolizers (IMs) and poor metabolizers (PMs) are 76.7%, 84.0% and 89.5%, respectively.

### Frequencies of key pharmacogenetic variants in SUPER-Finland

Predicted phenotypes and prevalence of *CYP2C9, CYP2C19, CYP2D6, DPYD, NUDT15, SLCO1B1, TPMT, UGT1A1* phenotypes in SUPER-Finland (total *n* = 9262) are described in Table 3. Observed minor allele frequencies of the variants used in the phenotype prediction are included in Supplementary Table 2. Based on *CYP2D6* imputation, *CYP2D6* gene duplication occurred in 8.5% (*n* = 791) and deletion in 2.7% (*n* = 247) of the SUPER-Finland participants. A total of 26 individuals carried the duplication in both homologous chromosomes and two individuals had both gene copies deleted. When these structural re-arrangements were combined with SNPs and translated to predicted CYP2D6 phenotypes, we observed that 6.6% (*n* = 607) of the participants were UMs, 62.7% (*n* = 5811) were NMs, 27.5% (*n* = 2545) were IMs and 3.2% (*n* = 299) were PMs.

**Table 3:** Predicted phenotype and prevalence of *CYP2C9, CYP2C19, CYP2D6, DPYD, NUDT15, SLCO1B1, TPMT* and *UGT1A1* in SUPER-Finland (total *n* = 9262).

Gene	Predicted Phenotype	Prevalence % (*N*)
*CYP2C9*	Normal	67.3 (6230)
	Intermediate (AS 1.5)	19.7 (1823)
	Intermediate (AS 1.0)	11.0 (1022)
	Poor	2.0 (187)
*CYP2C19*	Ultrarapid	3.8 (355)
	Rapid	24.3 (2254)
	Normal	39.7 (3676)
	Intermediate	28.7 (2656)
	Poor	3.5 (321)
*CYP2D6*	Ultrarapid	6.6 (607)
	Normal	62.7 (5811)
	Intermediate	27.5 (2545)
	Poor	3.2 (299)
*DPYD*	Normal	92.9 (8604)
	Intermediate (AS 1.5)	2.6 (242)
	Intermediate (AS 1)	4.4 (403)
	Poor (AS 0.5)	0.05 (5)
	Poor (AS 0)	0.09 (8)
*NUDT15*	Normal	96.3 (8915)
	Intermediate	3.6 (336)
	Poor	0.1 (11)
*SLCO1B1*	Normal	62.6 (5795)
	Decreased	33.6 (3110)
	Poor	3.9 (357)
*TPMT*	Normal	94.1 (8712)
	Intermediate	5.8 (539)
	Poor	0.1 (11)
*UGT1A1*	Normal	31.8 (2949)
	Intermediate	48.8 (4522)
	Poor	19.3 (1791)

*AS* activity score.

The prevalence of CYP2C19 UMs was 3.8% (*n* = 355). 24.3% (*n* = 2254) were classified as rapid metabolizers (RMs) and 3.5% (*n* = 321) as PMs, 39.7% (*n* = 3676) were NMs and the remaining 28.7% (*n* = 2656) were IMs. The predicted phenotypes for CYP2C9 were as follows: NM 67.3% (*n* = 6230), IMs with 1.5 activity score 19.7% (*n* = 1823), IMs with 1 activity score 11.0% (*n* = 1022) and PMs 2.0% (*n* = 187).

As the population history of Finland has created genetic subisolates within the country, we compared whether the prevalence of the pharmacogenetic phenotypes of CYP2D6 and CYP2C19 differ by recruitment center (Supplementary Tables 4, 5). For CYP2D6 UMs, the largest absolute difference was observed between Kuopio and Oulu (5.0% vs 7.7%; OR 0.63; 95% CI 0.47–0.83; *P* < 8 × 10^−4^).

A *NUDT15* variant classified as having no function (rs116855232-T) was enriched in Finland for 6.5-fold (*P* = 2.09 × 10^−181^) when comparing Finns and non-Finnish Europeans from GnomAD v2.1.1 (https://gnomad.broadinstitute.org) [30]. The minor allele frequency was 0.02 in GnomAD Finns as well as in SUPER-Finland (Supplementary Table 2) whereas it is 0.004 for Europeans in general.

A variant in *UGT1A1* gene encoding *6 haplotype (rs4148323-A) was enriched in Finland 22-fold (Supplementary Table 2). The minor allele frequency in Finns was 5% compared to 0.2% in non-Finnish Europeans.

## Discussion

We have shown that the *CYP2D6* copy-number can be reliably imputed for research purposes. The presented imputation method allows for imputation of the *CYP2D6* copy-number in large samples such as the UK Biobank and FinnGen. This in turn allows detailed cost-effective pharmacogenetic analyses, as these data sets have longitudinal drug prescription history available. As accurate inference of metabolic activity of CYP2D6 cannot currently be done from GWAS chip data alone, the copy-number imputation method creates new opportunities for development towards and research of individualized medicine.

For research use, the correlation between imputed and true copy-number is not required to be perfect if we are studying large cohorts, as even modest correlations will add information. To achieve similar statistical power for detecting an association using an imputed marker as opposed to using a directly genotyped one, the sample size must be increased by 5–13% for each 1% increase in imputation error [31]. Given that many pharmacogenetic studies conducted so far have a sample size below 2000 individuals, statistical power gained through increasing sample size to biobank scale (500k samples) would overcome the power loss due to inaccuracy in imputation. The imputation inaccuracy can be passed to statistical models for examples as allelic dosages, which range between 0 and 2. The closer the dosage is to a round number (0, 1, or 2), the more accurate the imputation result is. Other measures, such as posterior probability, have also been used [12]. As some pharmacogenetic variants are rare, imputation might be even more accurate than direct genotyping of the variant with a GWAS chip, as calling algorithms perform poorly on rare variants and thus they are usually excluded before imputation [32].

Genetic variation in *CYP2D6* has been described and compared between Finns and other European populations by earlier smaller studies. The Finns have been shown to have a high frequency of *CYP2D6* duplications and UM phenotypes compared with the ancestral European population [8]. Here, we show a high frequency of CYP2D6 UMs among subjects with a psychotic disorder throughout the country. *CYP2D6* genotype has an effect on metabolism of antipsychotics, such as risperidone and aripiprazole. This effect is further reflected on the therapeutic failure rate during risperidone therapy, suggesting that genotyping could be used to guide dosing decisions [3].

We confirm a high frequency of the *UGT1A1* variant rs4148323, also known as UGT1A1*6, in Finland. This variant has been linked to irinotecan toxicity in Biobank Japan [33]. According to GnomAD v2.1.1, the frequency of rs4148323-A in Europeans is only 0.2% but here we report, as seen earlier in a small sample size study [34], that the frequency in Finns is 4.2% which means an approximately 22-fold enrichment. The study concerning irinotecan-treated patients from Biobank Japan demonstrated that 51 out of the 330 subjects with normal UGT1A1 metabolism experienced adverse drug reactions (15%) whereas 8 out of 15 subjects (53%) with rs4148323-AA experienced an adverse drug reaction. As rs4148323-A is rare among European subjects, the drug trials involving irinotecan might not have captured the increased risk related to this genotype in Finns. Another *UGT1A1* variant, linked to decreased irinotecan metabolism is UGT1A1*28 or rs8175347 and the pertinent variant is also linked to Gilbert syndrome characterized by periods of intermittent icterus [35]. Since rs8175347 was not present in the SISu imputation panel, we used a proxy variant (rs887829) to estimate the frequency of UGT1A1*28 in Finns. The proxy variant is in strong linkage with rs8175347 in Finns according to LDlink [36] (r-squared 1.0) and also associated with Gilbert syndrome in FinnGen (https://www.finngen.fi/en/access_results). The proxy also shows an increased frequency among Finns compared with the rest of Europe, which leads to a high frequency of UGT1A1 poor metabolizers in Finland. This should be considered when planning to initiate irinotecan treatment for patients with Finnish ancestry.

*CYP2C19* genotypes have been shown to contribute to the metabolism of several antidepressants, clopidogrel, and proton pump inhibitors [5, 23, 37], for example. CYP2C19 genotype is associated with a failure of escitalopram treatment. Jukic et al. reported that 30.7% of CYP2C19 PMs discontinued escitalopram compared to discontinuation rates of 11.8%, 17.8%, and 28.9% in normal, rapid, and ultrarapid metabolizers, respectively [2]. Thus, subjects with an increased discontinuation rate make up 31.6 % of SUPER-Finland subjects, which is a clinically significant proportion of the patient base.

Recently, a new compound called siponimod, was introduced to markets in Europe and the USA for the treatment of multiple sclerosis [38]. The therapeutic dose of siponimod is dependent on the *CYP2C9* genotype. According to the manufacturer of siponimod, the drug is contraindicated in patients with the *CYP2C9* *3/*3 genotype (rs1057910-CC) and dose adjustment is needed for *2/*3 and *1/*3 genotypes. Thus, genotyping is necessary before initiating the drug. According to our results, the drug is contraindicated in about 0.5% of Finns and 11.2% require dose adjustments.

*DPYD* variants have a large impact on the safety of the chemotherapeutic agents capecitabine and 5-fluorouracil. Based on the phenotype prevalence observed here, 7.1% (*n* = 658) of individuals would require dose adjustment for these drugs according to the CPIC guidelines [24].

Variations in *NUDT15* were recently introduced in the CPIC guidelines to help estimate azathioprine dose for the treatment of Crohn’s disease, for example [25]. When determining proper azathioprine dose, *NUDT15* variants are interpreted together with *TPMT* variants. According to CPIC, the dose is determined by the *NUDT15* genotype or the *TPMT* genotype depending on which gene’s function is more severely affected. Thus, if the *TPMT* genotype is normal and the NUDT15 phenotype is an intermediate metabolizer, the dosing follows the recommendation for the NUDT15 intermediate metabolizer. When genotyping only *TPMT* (combined minor allele frequency of 5.6%) almost half of the individuals requiring dose adjustment based on the most up-to-date information are missed, while treating patients of Finnish ancestry.

The strength of our study is the large sample size, genotyped with a GWAS chip, which enabled us to estimate the haplotype structure in more detail, as compared with pharmacogenetic studies which have relied on genotyping of only a few variants. A limitation is that the sample was ascertained based on a previous diagnosis of psychosis and many patients were recruited from long-term treatment facilities and housing-units, which may skew the results towards patients who have had a sub-optimal therapeutic response, and thus divert the pharmacogenetic variant frequencies from the population mean. The BrePainGen study, from which the imputation panel was derived, included only women. However, this is not a problem because the *CYP2D6* gene is located in the autosome and the prediction of CYP2D6 phenotype from genotype does not differ between males and females. Part of the geographical differences between the recruitment areas in CYP2D6 and CYP2C19 phenotype frequencies of SUPER-Finland participants may also arise from trends in relocation based on available psychiatric care instead of natural habitation changes of Finnish population. Despite this, the geographical information can be used to inform local clinicians.

In conclusion, we demonstrate that despite complex structural variations in the *CYP2D6* locus, the copy-number can be computationally imputed. As the locus has consistently been shown to associate with drug concentrations, researchers are now able to expand these studies to disease outcomes using biobank data, for example. This will boost the development of individualized dosing algorithms and might eventually translate to improved patient care, especially in psychiatry. Over 20% of drugs listed by FDA as having pharmacogenetic information on their labels are used to treat psychiatric diseases. From these drugs, 69% are metabolized through CYP2D6 [39]. Additionally, we show that the bottle-neck effect contributes to the frequency of pharmacogenetic markers in Finland by demonstrating a high frequency of the decreased function variant UGT1A1*6. Further research should evaluate whether the observed phenomena affect the generalizability of the results of clinical trials across different ancestral groups.

## Supplementary information

Supplementary Table 1

Supplementary Table 2

Supplementary Table 3

Supplementary Table 4

Supplementary Table 5

## Data Availability

The data is available from THL Biobank when released from original study.

## Code availability

The computer code used in the analysis of this study is available from the corresponding author on reasonable request.
