# Supplementary material for: Implementation of CYP2D6 copy-number imputation panel and frequency of key pharmacogenetic variants in Finnish individuals with a psychotic disorder
Source: Pharmacogenomics J. 2022 Feb 23;22(3):166–72. doi: 10.1038/s41397-022-00270-y (PMC9151384; doi:10.1038/s41397-022-00270-y)
Supplement: Supplementary file 3 — Supplementary Table 3 [file 41397_2022_270_MOESM3_ESM.dotx]

Supplementary Table 3. Sensitivity, specificity, positive predictive value (PPV) and negative predictive value (NPV) for *CYP2D6* copy-number (CN) in SUPER-Finland.

*CYP2D6* CN=1 *CYP2D6* CN=2 *CYP2D6* CN=3

(deletion) (normal) (duplication)

Sensitivity (%) 88.9 89.4 98.6

Specificity (%) 96.6 96.4 94.2

PPV (%) 78.0 97.7 84.9

NPV (%) 98.5 84.1 99.5
