# Supplementary material for: Implementation of CYP2D6 copy-number imputation panel and frequency of key pharmacogenetic variants in Finnish individuals with a psychotic disorder
Source: Pharmacogenomics J. 2022 Feb 23;22(3):166–72. doi: 10.1038/s41397-022-00270-y (PMC9151384; doi:10.1038/s41397-022-00270-y)
Supplement: Supplementary file 4 — Supplementary Table 4 [file 41397_2022_270_MOESM4_ESM.docx]

Supplementary Table 4. Distribution of CYP2D6 metabolizers by recruitment area in SUPER-Finland.

% (n) UM NM IM PM n total

Helsinki 6.5 (152) 61.2 (1426) 28.4 (662) 3.8 (89) 2329

Tampere 7.5 (172) 62.1 (1428) 27.7 (637) 2.7 (61) 2298

Kuopio 5.0 (94) 65.6 (1238) 26.1 (493) 3.2 (61) 1886

Oulu 7.7 (134) 61.5 (1067) 27.8 (483) 3.0 (52) 1736

Turku 5.4 (55) 64.3 (652) 26.7 (270) 3.6 (36) 1013

UM, ultrarapid metabolizer; NM, normal metabolizer; IM, intermediate metabolizer; PM, poor metabolizer
